# Barnes on Diseases of Women

**Published:** 1878-11

**Authors:** 


*Barnes on Diseases of Women.* Second American from the Second and Revised London Edition.

This work has long been familiar to the American profession, though the first edition has been long exhaustive. The present edition of this work contains the addition of a chapter on the relations of Bladder and Bowel Disorders to the proper subject matter of the book. New Illustrations have been added, and when borrowed the source given from which it was taken. The Authors experience constitutes essentially the work, though numerous quotations are given and an index of Authors appended in connection with a very complete index of subjects. The illustration cuts are numerous, well made and instructive. His remarks upon the "*conditions indicating necessity for local examination*" are so suggestive that we take the liberty of quotation.

"There is nothing special in the mode of studying the diseases of women. Just as the ophthalmic surgeon is led to examine the eye because the patient complains of loss or disturbance of its function, or because he feels pain in it, or has some other subjective symptom referred to that organ, so by disturbances of function or some other subjective sign are we led to the discovery of disease of the sexual organs. When the function of an organ is disturbed, the *primâ facie* inference is that the organ itself which constitutes the mechanism by which that function is performed is out of gear. This is not indeed always absolutely true; because an impaired state of the blood, or disordered innervation, or derangement of another organ, may entail the functional disorder which arrests our attention. The genital organs are no exception to this proposition. The functions of the ovaries, uterus, or vagina may be seriously deranged by a state of anæmia or blood-poisoning, by disease of nervous centres, by disease of the heart, lungs, or liver. These functions may be even more seriously affected by mechanical pressure in continuous parts. Still the fact remains that we can hardly appretiate rightly or successfully treat these primary or correlated diseases if we do not take into careful consideration the state of the genital organs themselves. The general or distant affections require to be investigated and treated; but it is not safe to overlook the organs that may be secondarily involved.

It is needless to say that every woman who is ill and seeks advice does not suffer from disorder of the sexual system. She may labor under various constitutional disorders, and under disorders of parts of the body quite independent of the sexual system. On the other hand, general or local disorders may in their course react upon, and induce disorder in, the sexual system. And there are disorders of this special system, commencing in it, and in their turn reacting upon, and inducing disorder in, distant organs or in the general system. * * * * * * *

This is the question we have set before us: When a woman presents herself, complaining of certain symptoms, chiefly subjective, some, or perhaps none, referred to the pelvis, how are we to act? Will these subjective symptoms enable us to refer them to their cause, to establish a diagnosis, to give satisfactory indications for treatment? Hardly. We must, therefore, call to our aid hte objective signs; we must weight and determine the significance of these before wə can arrive at a conclusion at all precise or trustworthy as to the underlying pathological condition. The whole tendency of modern medicine is to subject every organ which manifests functional disorder to direct physical exploration, in order that it may solve the question presented obscurely by the subjective signs. The sound, the probe, the stethoscope, the the laryngoscope, the otoscope, the opthalmoscope, the various forms of speculum, are only so many contrivances for enabling us to project the senses of touch, sight, and hearing into the internal structures. In the case of the skin, all is at once exposed to direct observation; and, as Alibert remarked, we should be glad to have the same advantage in investigating and treating the diseases of the heart, lungs, liver, kidneys, and nervous centres. Why is it that the study of the pathology of the skin and of the eye is invested with such fascinating interest? Those who devote themselves wilh the greatest zeal and success to this study affirm that it is because the skin and the eye reveal their condition directly to the senses, and thus furnish not only positive objective signs which the patient can neither suppress nor misrepresent, but also because by this direct observation of the skin and eye they can read and follow, as on a map or on a telegraphic dial, the working of distant organs and of many affections of the general system. Here, then, we see how the reputed special practitioner, turning to account his special experience, often acquires an insight into general pathology denied to those who neglect the lessons they might read upon the visible organs.

This advantage we possess to a great extent of perfection in the case of the pelvic organs. It is by the proper use of this advantage that so great a degree of precision in knowledge, and of success in treatment of diseases of women, has of late years been attained.

And there is one property in a high degree characteristic of the instruments employed in the investigation of the diseases of women of such singular value that it ought to completely silence the objections at one time so passionately urged against them. It is this: the instruments have a thereapeutical as well as a diagnostic application; the speculum, for instance, revealing a lesion, often enables the surgeon at once to apply his remedy. Thus treatment follows upon the track of diagnosis, one sitting and one operation serving for both."

Its plain concise comprehensive style, commends the work very highly as a guide book in general practice, while yet the more important subjets in gynæcology have received quite full and complete consideration. It seems as a whole well adapted to the wants of students in medicine, as well as to active and experienced practitioners.

